# Electrodermal activity and heart rate variability for detection of peripheral abnormalities in type 2 diabetes: A review

**DOI:** 10.17305/bb.2022.8561

**Published:** 2023-10-01

**Authors:** Matej Žnidarič, Dominik Škrinjar, Alen Kapel

**Affiliations:** 1 Faculty of Medicine, University of Maribor, Maribor, Slovenia; 2 Faculty of Health and Social Sciences, Slovenj Gradec, Slovenia; 3 Alma Mater Europaea, Maribor, Slovenia; 4 Modus Medical, Maribor, Slovenia

**Keywords:** Diabetes mellitus (DM), diabetic foot ulcer (DFU), autonomic neuropathy (AN), electrodermal activity (EDA), heart rate variability (HRV)

## Abstract

Modern medicine exhibits an upward trend towards non-invasive methods for the early detection of disease and long-term monitoring of patients’ health. Diabetes mellitus and its complications are a promising area for implementation of new medical diagnostic devices. One of the most serious complications of diabetes is diabetic foot ulcer. The main causes responsible for diabetic foot ulcer are ischemia caused by peripheral artery disease and diabetic neuropathy caused by polyol pathway-induced oxidative stress. Autonomic neuropathy impairs function of sweat glands, which can be measured by electrodermal activity. On the other hand, autonomic neuropathy leads to changes in heart rate variability, which is used to assess autonomic regulation of the sinoatrial node. Both methods are enough sensitive to detect pathological changes caused by autonomic neuropathy and are promising screening methods for early diagnosis of diabetic neuropathy, which could prevent the onset of diabetic ulcer.

## Introduction

Diabetes mellitus (DM) leads to diabetic foot disease, which is a major health, social, and economic problem and is one of the leading causes of morbidity and mortality, especially in developed countries [1]. The number of people with DM was 463 million in 2019 and is expected to reach 700 million by 2045 [2]. One important cause of diabetic foot disease is diabetic neuropathy (DN), which effects on average 25% of all patients with DM and significantly impairs their quality of life [3]. The first part of the review focuses on the pathophysiological factors contributing to the development of diabetic foot disease as a result of chronic DM, and then elaborates on DN and peripheral vascular disease. The second part of the review article presents the feasibility and usefulness of skin electrodermal activity (EDA) and heart rate variability (HRV) measurements for the detection of diabetic foot disease in patients with type 2 DM.

## Materials and methods

The literature review was conducted via the most extensive medical literature databases (Medline, PubMed, ScienceDirect, and Google Scholar) for the timeframe 1975–2022. The used search terms were: “diabetic foot disease”, “diabetes mellitus”, “human sweat glands”, “diabetic neuropathy”, “peripheral vascular disease”, “peripheral arterial disease”, “electrodermal activity”, “heart rate variability”, and “diabetic neuropathy.” Any relevant review articles, randomized clinical trials, case reports, and case series found were included. In addition to the main research results obtained using the abovementioned databases, references of included resources were further examined and included, if suitable.

## Peripheral abnormalities in patients with type 2 diabetes

One of the most concerning complications of DM is the development of lower limb ulceration, which is a consequence of peripheral changes in patients with chronic DM [1]. The two main causes responsible for lower limb ulceration are ischaemia due to peripheral arterial disease and DN [4]. Studies show that patients with DM have a 25% lifetime risk of developing lower limb ulceration [5] and the annual incidence of patients with foot ulcers is 3% [6]. Lower limb ulceration is associated with complications that lead to amputation of the limb in 7.1% to 37.8% of the cases. As many as 40% of all amputations can be prevented by early detection of lower limb ulceration, before the initial symptoms of ulceration appear [7].

### Peripheral arterial disease

Peripheral arterial disease most commonly affects the tibial and peroneal arteries due to endothelial dysfunction and smooth muscle abnormalities as a result of a prolonged hyperglycaemic state. The development of peripheral arterial disease is closely linked to excessive vasoconstriction of the peripheral vasculature due to the pathological effect of hyperglycaemia on arachidonic acid metabolism. Under normal conditions, prostacyclin formation is the main result of arachidonic acid metabolism during functional hyperaemia. A reduced number of endothelial vasodilators leads to vasoconstriction. The production of thromboxane A2, which is produced in platelets by their own enzyme cyclooxygenase 1, decreases disproportionately with increasing functional hyperaemia [8]. Elevated levels of thromboxane A2, which acts as a vasoconstrictor and platelet aggregation agonist, lead to an increased risk of excessive blood clotting. Studies have shown elevated thromboxane A2 levels and decreased prostacyclin levels in urine and plasma of DM patients and animals, possibly due to altered arachidonic acid metabolism in the hyperglycaemic state [9, 10]. Studies in overfed and hyperglycaemic Zucker rats showed that the thromboxane receptor (TR) antagonist SQ-29548 partially restored the vasodilatory response to muscle contraction. This suggests that accelerated TR-dependent vasoconstriction is an important contributing factor to the impairment of functional dilation [11]. All the factors involved in the pathogenesis of peripheral arterial disease cause the lumen of the vascular wall to thin, ultimately leading to occlusion and subsequent ischaemia of the lower limbs. Lower limb ulceration occurs as one of the sequelae of peripheral vascular disease in patients with DM [12].

### Diabetic neuropathy

The concept of DN encompasses various clinical or subclinical forms of primary neuropathies that are characteristically present in patients with DM and for which no other cause can be identified. The most common complication of DN is diabetic polyneuropathy, the prevalence of which increases with the duration of DM and with higher levels of blood glucose. The development of DN is further accelerated by hypertension, smoking, age, and dyslipidaemia. The most common form of DN is distal symmetrical sensorimotor polyneuropathy, which accounts for 75%–80% of all DN. It is present in 7%–15% of patients at the time of detection of type 2 DM, and the incidence of DN in patients with DM increases over the years to as high as 50%. Distal symmetrical sensorimotor polyneuropathy plays an important role in the development of the diabetic foot disease and is characterized by symmetrical and neuronal length-dependent involvement of sensory and motor nerves. It occurs more frequently in the distal parts of the lower limbs and less frequently in the upper limbs. It is also characterized by proximal spread. A common form of DN that has a significant impact on the quality of life of patients with DM is autonomic neuropathy. It is characterized by a number of functional abnormalities, including abnormalities in the regulation of arterial pressure, heart rate, and abnormalities of the gustatory apparatus [13].

As DM progresses, asymptomatic neuropathy progresses to symptomatic polyneuropathy and, finally, to symptomatic autonomic neuropathy [13]. DN mainly impairs small C-type nerve fibers, which are part of the autonomic nervous system (ANS). However, regarding the timing of the onset of nerve fiber involvement, it should be noted that prolonged DM first affects the larger nerve fibers, which are myelinated and are primarily involved in motor function, vibration perception, and temperature changes. Small unmyelinated fibers become affected later [14]. Knowledge of the anatomical features of the peripheral nervous system may explain why the pathogenesis of DN differs from the pathogenesis of microvascular complications and why early DN first appears in the distal limbs. Peripheral nerves are covered by the perineurium, which is the connective sheath around the bundle of axons in the nerve. Only a few transperoneurial atria penetrate through the perineurium into the endoneurial area. The inadequate blood supply to the peripheral nerves and the inability to autoregulate make the system susceptible to ischaemia. The endoneural microcapillaries are in close association with endothelial cells on their inner surface. When the endoneural microcapillaries become damaged, they become permeable and consequently affect the endoneural tissue components. The permeable capillaries are mostly located in the ganglion with fenestrated capillaries. The nerve endings at the distal ends are thus directly exposed to areas not covered by the perineurium and are at higher risk of traumatic injury [15].

The reason why the distal regions are affected first in the process of DN is because ganglion cells have long axons covered with Schwann cells. The neuron body is relatively small compared to the large lengths of axonal neurites and, as a consequence, weak distal neurons are unable to transmit nutrients, various growth factors, and other signals [15]. Early metabolic changes that contribute to the onset and, at the same time, the progression of the complications of DM are regulated by “metabolic memory.” The development of vascular complications of DM is influenced by an inherited predisposition that triggers inflammatory events before the negative effects of hyperglycaemia become apparent. Inflammation and hyperglycaemia trigger a cascade of events affecting cellular proteins, gene expression, and cellular receptor expression in the vascular endothelium, resulting in progressive pathological vascular complications of DM [14].

Finally, the pathological signs of DM are also manifested in sweating. Sweating is controlled by the central nervous system and ANS via the neurotransmitters acetylcholine (ACh) and norepinephrine. Unlike ACh, norepinephrine plays only an indirect role in sweating [16]. Autonomic neuropathy results in hypohidrosis or reduced sweating [17]. As a consequence of long-term hyperglycaemia, the glucose metabolic cascade leads to peripheral nerve damage with increased formation of glycation end products, excessive cytokine release, activation of protein kinase C, and excessive oxidative stress, as well as accelerated formation of products of the polyol metabolic pathway [15]. Involvement of autonomic nerve terminals inhibits the release of neurotransmitters in the sweat gland area and, therefore, despite activation of the cholinergic nervous system, the amount of sweat secreted does not increase [17].

A prominent consequence of DN is the development of diabetic foot disease. Prolonged hyperglycaemia plays an important role in the development of DN, causing a number of metabolic abnormalities [18]. One of the pathophysiological mechanisms thought to play a key role in the development of DN is the polyol pathway [19]. The hyperglycaemic state leads to increased activity of the aldose reductase and sorbitol dehydrogenase. The enzymatic catalysis results in the conversion of intracellular glucose to sorbitol and fructose. Hyperglycaemia and associated oxidative stress further contribute to improper glycation of neuronal proteins and inappropriate activation of protein kinase C, ultimately leading to ischaemia. Thus, DN results in motor, autonomic, and sensory impairment of the nervous system. Disrupted innervation of the intrinsic muscles of the foot leads to a disproportion between flexion and extension of the affected foot, which causes anatomical deformities and further leads to progressive disruption of the skin tissue and ulceration [4]. Autonomic neuropathy, for instance, leads to reduced function of sweat glands and sebaceous glands, resulting in dry skin that becomes more susceptible to cracking and infection. Due to the loss of sensory function in the foot, the patient cannot feel the feet. Inadequate shear forces and excessive pressure on a certain part of the foot lead to ulceration, which is often overlooked due to the sensory impairment, and as a consequence, the ulcer site may become infected, leading to amputation of the limb [1].

### Non-invasive detection of diabetic neuropathy

The diagnosis of DN is made by excluding other causes based on medical history, clinical examination, laboratory, and instrumental tests. Assessment of sudomotor function can be performed by quantitative sudomotor axon reflex (QSAR), thermoregulatory sweat test with a colored sweat indicator, and by measurement of the sympathetic skin conductance response (SCR) [20]. The QSAR test is used to assess postganglionic sympathetic-cholinergic sudomotor function by measuring the amount of sweat produced over time. The sweat glands are stimulated by iontophoresis with a cholinergic agent and then the moisture concentration is measured with a hygrometer. The sweat thermoregulatory test assesses the function of central and peripheral sympathetic sudomotor pathways from the central nervous system to the sweat glands. As the room temperature rises, the subject’s blood and skin temperature increases. Sweating is assessed using a color indicator [21].

### Physiology of the human sweat glands

Sweating is a thermoregulatory response in many animals and an important evolutionary advantage in humans. The ability to sweat can be attributed to the gradual migration of our distant ancestors from forests to warmer areas. Human sweat differs from that of other animals in that it contains a higher proportion of water. Through evolution, humans have developed unique sweat glands, unlike other animals, whose sweating is facilitated by apocrine glands. The latter, unlike the sweat glands, secrete mainly a higher proportion of lipids and proteins [22]. The measurement of SCR is intrinsically linked to the function of the sweat glands, so in a first step, we present their function and resuscitation in a narrower sense. Sweat glands are exocrine glands that secrete directly onto the surface of human skin. The human body has approximately three million sweat glands and their density is highest on the arms, legs, and trunk [23]. The average number of sweat glands per cm^2^ on male skin is 233 on the palms, 620 on the forearms, 360 on the forehead, 120 on the thighs, 497 on the sole, 155 on the dorsal side of the foot, and 57 on the shins [24, 25]. Most human sweat glands are eccrine, meaning that their sweat does not contain traces of cytoplasm from glandular cells (as in sebaceous glands, for example). A synonym for eccrine secretion is merocrine secretion, where secretion is by exocytosis and the cell remains intact. Sweat gland activity is therefore crucial in the homeostatic process of thermoregulation [25].

Sweat gland activity is controlled by the ANS. Human skin is covered by several efferent autonomic fibers, which consist of sympathetic fibers that innervate the secretory segment of the eccrine glands and arrector pili muscles, which allow vasoconstriction. The secretory role of the sweat glands is facilitated by the postganglionic sympathetic nerve fibers, which are composed of unmyelinated C-type fibers. Some of these extend to the dermal part of the glandular duct. The postganglionic sympathetic fibers leave the sympathetic part of the spinal cord via the gray ramus communicans and merge to form the mixed spinal nerve, which contains all the motor and sensory fibers going to the periphery and back. The sensory fibers provide surface sensation to the skin and form characteristic plexuses in the subcutaneous tissue. The spinal cord segments T2 to T8 provide skin resuscitation of the upper limbs, the segments T1 to T4 resuscitate the skin of the facial region, and the segments T10 to L2 resuscitate the skin of the lower limbs. When sympathetic resuscitation is involved, noradrenaline is usually the main peripheral neurotransmitter. Eccrine gland cells are an exception because their main neurotransmitter is ACh, on which the amount of sweat secreted depends. The myoepithelial cells of the sweat glands express numerous muscarinic ACh receptors of the M2–M5 type on their surface, and the acinar cells of the sweat glands express muscarinic ACh receptors of the M1, M3, and M4 type [26]. The secretory part of the sweat glands is surrounded by a very dense plexus of sympathetic fibers. The exact mechanism of sweat secretion is yet unknown, but it is thought that myoepithelial cells surrounding the secretory segment of the sweat gland respond to elevated amounts of ACh with contraction [27]. Sweat glands in palmar and plantar sites play an important role in thermal perspiration [23]. At these sites, sweating starts exclusively at high temperatures, whereas at room temperature the glands secrete minimal amounts of sweat [28].

The secretory segment of the sweat gland, surrounded by a layer of myoepithelial cells, is composed of light and dark secretory cells. The light cells produce the liquid part of the sweat, while the dark cells secrete mucin, which is thought to play a protective role in the lumen of the sweat gland. Sweat is composed mainly of water, but also contains ions, lactate, and urea. The ionic composition varies according to the individual, their adaptation to heat, exercise and perspiration, the specific conditions (sauna, etc.), and the duration of perspiration [29]. Primary human sweat contains approximately 147–151 mM Na (sodium), 123–124 mM Cl (chlorine), 5 mM K (potassium), 10–15 mM HCO3 (bicarbonate), and 15–20 mM lactate anion [27]. The composition of primary sweat is thus comparable to that of extracellular fluid [30]. The amount of NaCl in sweat decreases after sweat reaches the skin surface during sweating. This phenomenon can be explained by the action of the dermal ducts, whose main role is to reabsorb electrolytes and to prevent excessive NaCl loss during sweating at higher temperatures [27]. Sweat is not secreted continuously but in pulsations between 12–21 Hz [31], which are responsible for the rhythmic contractions of the myoepithelium [32].

In addition to sweating, there are three physiological variants of sweating: emotional sweating, drug-induced sweating, and sweating due to the axonal reflex (Table 1) [33]. Emotional sweating (also psychological sweating) is one of the forms of sweating that becomes more pronounced with intense irritation and stress and is most pronounced in the palmar and plantar regions, as well as in the axillary, genital, and forehead regions [28]. The higher density of sweat glands results in a greater amount of sweat secreted in these areas [34]. Psychological stress triggers a rapid response of the sympathetic nervous system, which in turn increases the amounts of catecholamines, including adrenaline and norepinephrine, resulting in an accelerated heart rate, an increase in respiratory rate, and an increase in blood pressure. Sweating on the palmar and plantar areas of the skin is already present in newborn infants at 10 days of age. In the axilla area, however, psychological sweating does not start to appear until the onset of puberty. The exact origin of the psychological sweating is unknown, but it is thought that the sweating trigger centers are in the frontal cortex, hippocampus, and amygdala. The latter is thought to play a major role in the response to acute psychological stress [35]. Psychological sweating is a mechanistic substrate for indirect measurement of ANS function, by changes in the electrical conductivity of the skin, which is addressed in the next section.

**Table 1:** Brief description of the physiological variations of sweating

**Physiological sweating**	**Brief description**
Emotional sweating	A form of perspiration that becomes more pronounced with severe irritation and stress and is most pronounced in the palmar and plantar areas, as well as in the axillary, genital and forehead areas.
Drug-induced sweating	Drugs that are mainly cholinergic stimulants (pilocarpine, physostigmine, methacholine, and others) increase the amount of sweat secreted.
Sweating due to axon reflex	Intradermal application of acetylcholine or epinephrine causes sweating at the site of application.

## Electrodermal activity (EDA)

In the following part of the article, we present the physiological background of EDA in greater detail. The dermis and hypodermis, which are well supplied with blood and interstitial fluid, have a relatively good electrical conductivity, which can vary at the expense of blood flow. The stratum corneum does not contain living cell membranes but only keratinized cells that maintain a diffusion balance between the inside and the outside of the dermal epithelium. The stratum corneum acts like a sponge, transferring fluids from the inner layers of the skin to the surface. Increased perspiration increases its hydration, resulting in tonic or slow phase changes in skin resistance. A dry stratum corneum, either due to aging of the skin or due to spontaneous reabsorption of water in the dermis, causes an increase in the tonic skin resistance [36]. Current knowledge suggests that the conductivity of the stratum corneum is more dependent on its electrolyte content than on its moisture content. An electrolyte is a substance that, when dissolved in a polar solvent, such as water, forms an electrically conductive solution. The dissolved electrolytes are uniformly distributed in the polar solvent. If an electric potential is created in the solution, the cations are oriented in the direction of the electron-rich electrode and the anions are oriented in the direction of the opposite electron-deficient electrode. The movement of cations and anions in opposite directions generates an electric current. The wetting of the stratum corneum by sweat has a much greater effect on its conductivity than the imperceptible perspiration that passes through the epidermis [37].

The resistive properties of the skin and the sweat gland system can be described by parallel and series resistors. The first variable resistor is the stratum corneum, the fixed resistor is the epidermal barrier, the third resistor are the sweat glands, and the fourth resistor are the lower layers of the epidermis, dermis, and hypodermis with their relatively low resistivity [27].

The SCR is a transient change in the electrical resistance of the skin, triggered either spontaneously or reflexively by several internal (intrinsic) or external stimuli. Stimuli that activate the sympathetic nervous system in humans include deep breathing, coughing, loud noises, electric shocks, and others. More specifically, the SCR is a somato-sympathetic reflex with spinal, bulbar, and suprabulbar components, but the origin and course of the reflex itself are not defined in detail. The brain centers that influence the SCR are the ventromedial prefrontal cortex, the anterior cingulate cortex, the parietal gyrus, the insula, the amygdala, and the dorsolateral prefrontal cortex [38]. Their importance in the genesis of the SCR is complex and beyond the scope of this article. The amygdala is thought to play a key role in the genesis of SCR and is the first element of the neural cues for the genesis of SCR. The amygdala connects the sensory association cortices with the preganglionic elements of the sympathetic nervous system that innervate the sweat glands, which are responsible for the genesis of SCR [39].

SCR can be quantified by measuring the EDA, which measures the level of sudomotor function in a simple and non-invasive way and is also a sensitive indicator of emotional arousal and attention. EDA was first defined by Johnson and Lubin in 1966 [40]. A change in EDA is caused by the sympathetic cholinergic sudomotor response, which alters skin resistance due to altered sweat gland activity. Increased sweat gland secretion increases the electrolyte and water levels at the skin surface, which can be detected as increased skin conductance (SC) by EDA measurement [37, 41]. With the development of methods to measure SC, the same measurements have been referred to by other terms, such as SCR, Galvanic Skin Response (GSR), and Skin Conductance Level (SCL). Today, the term EDA is in common use [27].

There are endosomatic and exosomatic methods of measuring EDA. The endosomatic method of EDA measurement is a much less studied method and is based on the measurement of the properties of the electrodermal system that are the result of active events within the system itself. It is assumed that the electrical energy originates in polarizing membranes in the skin [27]. The endosomatic method of EDA measurement is less commonly used in practice, so in the following, we focus on the exosomatic method of EDA measurement.

**Figure 1.**
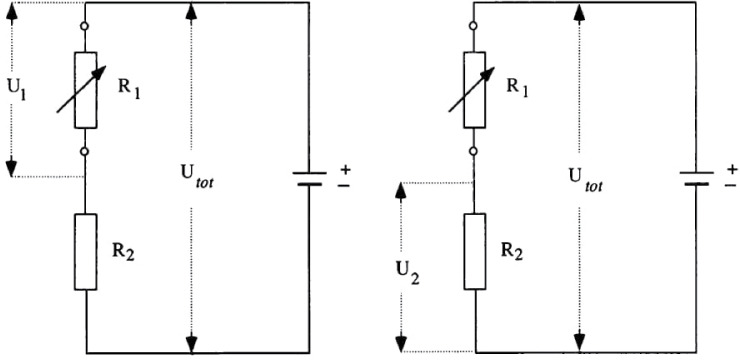
**The exosomatic principle of EDA measurement.** Electrical circuits showing the constant current (left figure) and constant voltage (right figure) methods of EDA measurement. R_1_ and R_2_ represent fixed reference resistors. A detailed description is given in the text. EDA: Electrodermal activity.

Most devices that measure EDA are based on an exosomatic approach. In this procedure, a constant external electric current or voltage difference is delivered through two electrodes placed on the skin. Exosomatic devices measure a modulated current or voltage depending on whether the source is an electrical voltage (most commonly) or an electrical current to determine the SC value, with respect to Ohm’s law. Figure 1 shows in more detail the electrical circuit of the exosomatic EDA measurement method. Two electrodes are placed on the skin and connected in series with a system reference resistor. A constant voltage—U_tot_—is applied to the voltage source. The active changes in the electrodermal system are considered to be fluctuations in the measured partial voltages. The left circuit in Figure 1 shows the situation when the source for determining SC is an electric current. The voltage is measured across resistor R_1_, the value of which represents the skin resistance. The measured voltage, U_1_, is in the same ratio to U_tot_ as R_1_ is to R_tot_. R_tot_ is the sum of the resistances R_1_ and R_2_, which implies:

$$\frac{U_{1}}{U_{\text{tot}}}=\frac{R_{1}}{R_{1}+R_{2}}$$

Considering Ohm’s law, which for a given electric circuit gives the proportionality between the electric current I_tot_ through an electric resistor and the voltage across it ($I_{\text{tot}}=\frac{U_{\text{tot}}}{R_{\text{tot}}}$), where R_tot_ represents the total resistance, the above equation can be rearranged to:

$$I_{tot}=\frac{U_{\text{tot}}}{R_{\text{tot}}}=\frac{U_{\text{tot}}}{R_{1}+R_{2}}$$

When the system is calibrated so that the fixed reference resistor R_2_ is significantly larger than the variable skin resistance R_1_, the total I_tot_ current is determined via resistor R_2_. This results in SC measurement system with a constant electrical current. The right circuit in Figure 1 shows the situation when the source for determining SC is an electrical voltage. The voltage is measured at resistor R_2_, which in this case is a fixed resistor. The measured voltage U_2_ is in the same ratio to the total voltage input U_tot_, as R_2_ is to R_tot_, which is defined as R_1_ + R_2_, hence:

$$\frac{U_{2}}{U_{\text{tot}}}=\frac{R_{2}}{R_{1}+R_{2}}$$

When the equation is rearranged by multiplying the both sides by U_tot_, we get:

$$U_{2}=U_{\text{tot}}\frac{R_{2}}{R_{1}+R_{2}}$$

When the system is calibrated so that the fixed reference resistor R_2_ is much smaller than the variable skin resistance R_1_, it follows that the total current I_tot_ is no longer constant: the resistor R_2_ can be neglected and the value of the electric current can be considered to increase at the expense of the decrease of the skin resistance R_1_ and vice versa. Since the voltage drop across R_2_ is negligible, the total voltage U_tot_ is applied to the skin. This results in an EDA measurement system with a constant electrical voltage [27].

### The principle of EDA measurement

**Figure 2.**
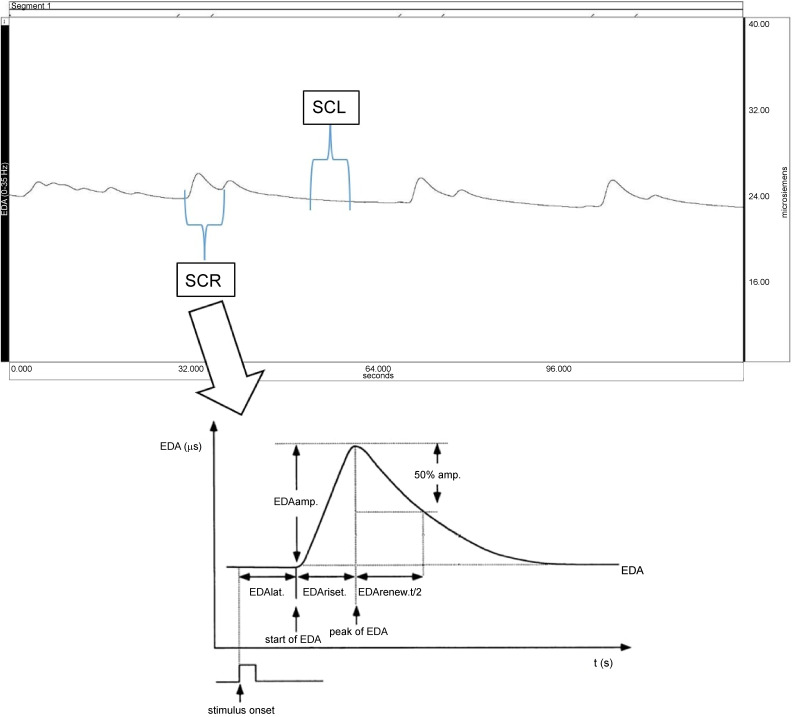
**The upper part of the figure shows the continuous EDA measurement curve with the BIOPAC^®^ meter, while the lower part of the figure represents the ideally measured skin SCR with all the parameters presented in the text.** EDA_amp._: Maximum SCR amplitude; EDA_lat._: Delay time from stimulus onset to the first significant change in skin conductance value; EDA_riset._: Time from the onset of the electrodermal response after the stimulus to the maximum measured skin conductance amplitude on the graph; EDA_renew.t/2_: Time required for the measured skin conductance value to decrease to 50% of the maximum measured SCR after the onset of the applied stimulus; 50% amp.: Half the maximum measured SCR value; EDA: Electrodermal activity; SCR: Skin conductance response.

Professional academic measurement devices allow accurate evaluation of EDA [42]. The continuous EDA measurement curve and the ideally measured skin EDA with all parameters are presented in Figure 2. The EDA curve distinguishes two SC components, namely, the SCL, which represents the SCL over a long-time interval at rest, and the SCR, which reflects the rapid changes of the SC after a triggered stimulus that activates the human ANS and only covers a small fraction of the whole EDA complex. Siemens is the unit of conductance, while ohm is the unit of resistance. 1 siemens represents 1/ohm. EDA_lat._ in Figure 2 represents the delay time from stimulus onset to the first significant change in the SC value on the graph between 1–3 s after the stimulus is delivered. EDR_amp_. represents the peak value of the measured SC amplitude, which represents the peak of the electrodermal response after the stimulus. EDA_riset_. is the time from the onset of the electrodermal response after the stimulus to the peak of the measured SC amplitude on the graph. In certain cases, we are also interested in the EDA_renew.t./2_, which represents the time required to reduce the measured SC value to 50% of the maximum measured SCR after the onset of the applied stimulus. We may also be interested in the 50% and 63% of the maximum measured SC value after a single stimulus is triggered (50% amp., 63% amp.) [42].

**Figure 3.**
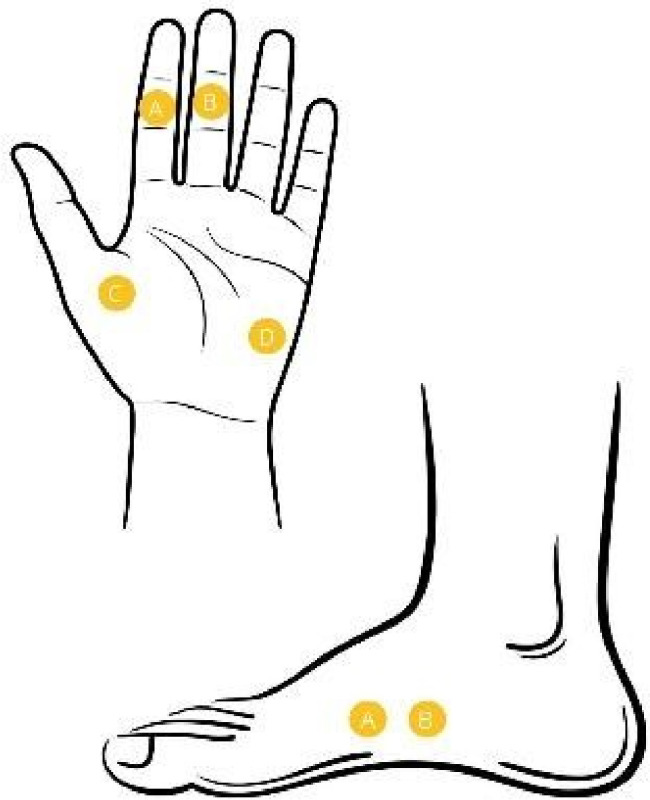
**The most common EDA measurement sites.** EDA: Electrodermal activity.

Non-polarizing electrodes are used to measure EDA and are glued to the palm, soles, and inside of the wrist, or to the area of the dermatome controlled by sensory neurons from the spinal ganglion (Figure 3). It is also advisable to place the electrodes on areas of the skin with high sweat gland activity under the influence of the cholinergic nervous system, as this also results in a greater change in SC when the appropriate stimulus is applied (soles of the feet, palm of the hand) [27]. Often considered the standard method of EDA measurement, is the exosomatic direct electric current EDA measurement, where a constant voltage of 0.5 V or a constant current not exceeding 10 microA/cm^2^ is applied via electrodes to the measurement site, which is lubricated with a neutral NaCl gel prior to the measurement. The skin where the electrodes are applied shall not have been previously cleaned with water or soap [17].

### Early changes in EDA in patients with type 2 diabetes

To date, several authors of scientific studies have investigated the impact of DN on altered EDA_amp._ values. Abnormal outcomes of SCR measurements have been measured in 66%–83% of DM patients [13]. Braune and Horter showed already in 1995, in a large prospective clinical study on 100 DM patients, that skin EDA varies according to the degree of DN. The measured EDA_amp._ was lower in at-risk patients than in healthy controls, suggesting that the SC of the feet and hands is lower in patients with DM, mainly due to lower volumes of sweat secreted [43]. Results from a 2009 study where EDA was measured in three groups of people (healthy young individuals, elderly people, and DM patients) showed that DM patients had, on average, the lowest measured EDA_lat._ values [44]. The most responsive sites for SCR measurement are the foot, toes, fingers, and shoulders, while the least responsive sites are the hand, back, armpit, and thigh [45]. The distal toe site is the most sensitive site for detecting DN by electrodermal measurement, as the first signs of neuropathy start to appear in the most distal parts of the limbs. Thus, EDA values were studied in 47 DM patients diagnosed with DN and compared with a control group of 24 healthy individuals. SCR values could be measured in all control subjects, while the amplitude of the EDA_amp._ response was very reduced in 66% of the patients and SCR values could not be measured in the feet in 27.7% of the patients. They also found that the absence of SCR was more prevalent in patients with known ANS dysfunction (*P* < 0.05), despite being asymptomatic [46]. EDA measurements were also performed in children with insulin-dependent DM. EDA was compared in 28 healthy children and in a group of 8 children with insulin-dependent DM without proven ANS impairment. The time from stimulus onset to the maximum measured SCR amplitude—EDA_lat._—was, on average, longer than the measured EDA_lat._ values in the group of healthy children [47]. Although many studies in the field of electrodermal measurement describe the usefulness of the EDA measurement method as a screening test for the detection of insulin-dependent DM, some studies measured reduced EDA_lat._ values. The differences in measured EDA_lat._ values were statistically significantly different between the feet of DM patients who had a high probability of developing DN. It should be noted that the studies did not follow the patient’s health status up to the point at which they developed DN. In many DM patients, the early symptoms of autonomic dysfunction are masked and non-specific. This suggests that in patients with long-standing DM, signs of DN are not present from the outset but may already be detectable as a change in the SCR (48–51). A more recent study presented a protocol for performing EDA measurements considering the patient’s emotional state, the volume status during the test, and the body position during the measurements, where the patient sits in a semi-sitting position with legs extended, does not move during the measurements, and monitors a white dot on a black screen throughout the measurements. It has been pointed out that it is useful to compare the differences in mean SC values between the left and right foot and, consequently, to determine which limb is more affected by DN [52].

## Heart rate variability (HRV)

In recent decades, HRV analysis has become established for the assessment of autonomic system activity, allowing non-invasive assessment of the sympathovagal response to the sinoatrial (SA) node [53, 54].

HRV represents the variability in the time intervals between successive heart beats and reflects the interactions between multiple regulatory systems in the human body [55]. Among others, HRV reflects the functioning of the ANS, changes in blood pressure and gas exchange, the functioning of the digestive system, the heart, and vascular tone, which refers to the diameter of blood vessels that control blood pressure [56]. The human heart is not a metronome, and the oscillations of a healthy heart are anything but linear and simple. HRV values vary depending on age, method and length of measurement, sex, and baseline HRV before measurement. Optimal HRV values, according to the above factors, are related to human health, self-regulation of physiological processes in the body, adaptability, and resistance to the possible initiation of a pathophysiological process in the human organism [57].

The most common methods for measuring HRV are electrocardiography (ECG) and photoplethysmography (FPG).

### Physiology of heart rate regulation

To facilitate the understanding of HRV, the following sections introduce the action of the ANS on the cardiac muscle, the influence of parasympathetic and sympathetic activity, and the basic principle of ECG measurement, which is the basis for understanding HRV. HRV expresses the variability of heart rate and can be used to assess the function of the ANS. The heart functions autonomously, with its intrinsic frequency determined by the rate of spontaneous diastolic depolarization in the SA node. The spontaneous diastolic depolarization is due to the flow of potassium ions through the so-called funny channels, which are located on the membranes of the cells of the SA node. The influence of the SA can modify this intrinsic frequency. Thus, the parasympathetic branch lowers the frequency due to the binding of Ach to M2-type muscarinic receptors, which triggers a decrease in heart rate. Conversely, the sympathetic branch increases the frequency due to binding to β1 adrenergic receptors [58, 59].

**Figure 4.**
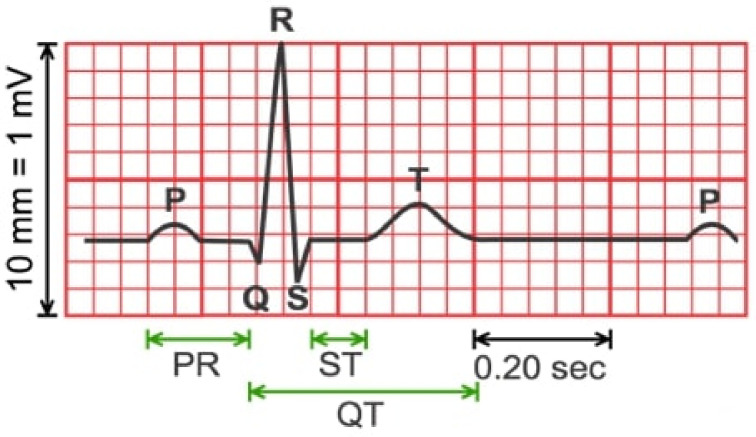
**A standardized ECG recording.** ECG: Electrocardiography.

### The principle of measuring HRV

The ECG signal is measured using electrodes attached to the skin of the body. The ECG measuring device measures the signal and displays it as a two-dimensional standardized recording of the signal. An example of an ECG is shown in Figure 4. 1 cm of graph height corresponds to a voltage of 1 mV. In healthy people, the features on an ECG recording are similar. The *P* wave represents atrial depolarization (lasting up to 0.10 s), the PQ interval represents atrioventricular conduction time (lasting between 0.12 s and 0.20 s), the QRS complex represents ventricular depolarization (lasting between 0.10 s and 0.11 s), and the T wave represents ventricular repolarization. The QT interval is measured from the beginning of the Q wave to the end of the T wave. In medical practice, electrodes are connected to different points on the surface of the body to measure the potential differences between the electrodes. The most common way of connecting electrodes, pioneered by Einthoven in 1903, is to the right arm (R), left arm (L), and left leg (F). The voltages recorded by Einthoven are U1 ═ ΦL − ΦR, U2 ═ ΦF − ΦR, U3 ═ ΦF − ΦL, where U3 ═ U2 − U1 [60].

HRV can be calculated from the ECG recordings using linear and, more recently, non-linear analyses. Linear analyses of HRV are based on Euclidean geometry and statistics. Non-linear analyses are based on the mathematics of complex dynamics and Mandelbrot fractal geometry [61]. For linear analysis, we focus on time-indicator analysis and frequency analysis. Time-domain indicators reflect the magnitude of the change in heart rate and frequency-domain indicators reflect the rate of change in heart rate. Time-domain HRV indices are mostly obtained from long, 24-h ECG recordings and are divided into two groups. In the first group, the indices are obtained by monitoring individual NN intervals (the N tooth is the R tooth in the ECG display, which is due to depolarization of the sinus node, and the NN interval is the interval between two adjacent N tooth). The second group includes indices obtained by observing the differences between NN intervals. Time-domain indicators reflect parasympathetic activity.

The following indicators are the most used in practice:

- NN intervals (the mean of the NN intervals),
- SDNN (standard deviation of all NN intervals),
- SDANN (standard deviation of the mean NN intervals calculated from 5-min intervals),
- RMSSD (square root of the root mean squared difference between two adjacent NN intervals),
- SDNN index (mean of the standard deviations of all NN intervals obtained from 5-min intervals),
- SDSD (standard deviation of the difference between two adjacent NN intervals)
- pNN50 (frequency of adjacent NN intervals that differ by more than 50 ms) [53].

For the analysis of shorter ECG recordings of around 5 min, we mainly use frequency-based methods, which are based on the observation of groups of sinusoidal waveforms of different frequencies and amplitudes, obtained by decomposing a sequence of NN intervals. In frequency analysis, we monitor the total power of the spectrum in a frequency interval <0.4 Hz, which can be further subdivided into individual spectral regions and individually calculate their power:

- a high frequency (HF) component between 0.15 and 0.4 Hz, representing the vagus activity,
- a low frequency (LF) component between 0.04 and 0.15 Hz, which is an indicator of sympathetic activity,
- a very low frequency (VLF) component between 0.003 and 0.04 Hz,
- an ultra-low frequency (ULF) component in the frequency interval <0.003 Hz.

For observing the functioning of the ANS, the most useful linear analysis of HRV is the power ratio of the low- and high-frequency component (LF/HF), which represents the sympathetic-vagal balance [61, 62].

### Early HRV changes in patients with type 2 diabetes

Peripheral neuropathy is a relatively common complication in patients with DM, affecting 33.5% of patients and leading to an increase in morbidity and mortality. Studies measuring HRV in patients with DM are numerous, but the results are rather inconsistent [63]. Diabetic polyneuropathy also involves simultaneous impairment of both branches of the ANS, which is termed diabetic autonomic neuropathy [53]. A more recent study has shown that isolated peripheral neuropathy without associated DM complications does not have a statistically significant effect on the lower HRV values measured in patients with type 2 DM but becomes significant when the patient with DM has multiple associated complications—diabetic retinopathy and nephropathy [64]. Patients with DM without signs of diabetic polyneuropathy have significantly lower absolute values of linear markers [53].

Islam et al. used a photoplethysmography-based device to measure HRV in patients with type 2 DM. They aimed to examine the following parameters from the plethysmography pulse waveform—mean heart rate, SDNN (standard deviation of all NN intervals), RMSSD (root mean square difference between adjacent NN intervals), and total power. The parameters from the frequency domain of the measurements were VLF component (0.01–0.05 Hz), LF component (0.04–0.15 Hz), and HF component. The results presented that male DM patients with distal peripheral neuropathy had statistically significant lower HF and LF powers compared with male patients without distal peripheral neuropathy. On average, male patients with type 2 DM had lower HRV values [63]. HF power is proportional to parasympathetic activity and the elevated LF/HF ratio in men is most likely due to an imbalance between the sympathetic and parasympathetic nervous systems. Higher values of the LF/HF ratio indicate increased sympathetic activity, while lower values indicate increased vagus activity [65]. Thus, a study by Islam et al. showed that parasympathetic activity in DM patients decreases with increasing severity of distal peripheral neuropathy [63]. It is interesting to note that healthy female subjects have higher parasympathetic activity than males [66].

In the second half of the 20th century, several authors of scientific studies noted significant changes in HRV in patients with DM. These studies have shown that HRV can be used as an unbiased assessment of the ANS in patients with diabetes [67–69], with lower HRV measurements in patients with neuropathy onset [68, 70, 71]. Since the initial observations of this phenomenon, reduced HRV has been considered an early and asymptomatic phenomenon at early onset of DM. Reduced HRV correlates with impairment of parasympathetic innervation of the vagus nerve, the longest autonomic nerve, which manifests as impairment of peripheral somatic nerve fibers in the case of diabetic sensorimotor polyneuropathy [72]. Among others, HRV during deep breathing is one of the cardiovascular ANS reflex tests used as the gold standard in the detection of cardiac autonomic neuropathy [73]. Osterhues et al. aimed to determine whether HRV measurements in patients with insulin-dependent diabetes can distinguish peripheral from autonomic neuropathy and to identify which part of the central nervous system is affected at the earliest stage of neuropathy, before the characteristic signs of autonomic dysfunction appear. The results showed that 24-h HRV measurements could detect neurological impairment resulting from an underlying parasympathetic nerve involvement [70]. HRV was also lower in children with insulin-dependent diabetes compared with healthy controls. Similar results were reported by Akinci et al. [74]. Orlov et al. hypothesized that HRV may be an independent biomarker of early diabetic sensorimotor polyneuropathy. They attempted to compare the diagnostic performance of DN using HRV measurements and established gold standard diagnostic tests in 89 US patients with type 1 DM. They identified a positive correlation between lower HRV measurements (lower HF and LF values and elevated LF/HF ratio) and higher severity of diabetic sensorimotor neuropathy. They also concluded that HRV values represent markers of early and late complications of neuropathy by comparing the measured values with validated tests of small and large nerve fiber activity [75]. However, no study to date has investigated whether low HRV values and their change over time can predict the onset of sensorimotor polyneuropathy in patients with DM.

Other common clinical conditions that are linked to a reduced HRV include coronary heart disease (CHD) and atherosclerosis. In a study conducted by Liao et al., it was found that a decrease in HRV is associated with the development of CHD in individuals with diabetes. The study concluded that HRV is a significant factor in CHD risk among individuals with DM and is mostly independent of other markers of glucose metabolism impairment. The findings of the study suggest that an impaired cardiac autonomic control contributes significantly to the risk of CHD in individuals with DM [76].

Similarly, a review of 15 studies by Schiwe et al. [77] found that children and adolescents with asthma have a lower HRV and a lower level of sympathetic modulation. The review concluded that these differences were significant when compared to healthy individuals and were revealed by long-term low-frequency measurements. Lower HRV has also been linked to several other clinical conditions, including psychiatric ones, such as anxiety disorders [78].

### Monitoring accuracy and limitations of EDA for diabetic neuropathy and peripheral arterial disease detection

Exosomatic EDA devices are the most commonly used, where the skin is stimulated by an external constant current or voltage source through electrodes. These sources can either be direct current (DC) or alternating current (AC). A major issue in EDA measurement is the electrode polarization caused by the constant source. In exosomatic DC-source devices, the electrodes become polarized as they carry a DC, acting as an anode and cathode. Reusable electrodes, although more expensive, are available commercially, unlike disposable AgCl electrodes with a thin silver chloride layer. However, it has been observed that even with low DC, the charge in reusable electrodes can become high enough to remove part of the AgCl layer at the cathode, increasing the layer at the anode, resulting in significant voltage bias levels after extended use [79].

One of the major challenges in interpreting the SCL and Nonspecific SCRs (NSSCRs) is their high variability across individuals. Periodic changes in the background SCL, such as DC shifts, may also be crucial if they occur in conjunction with specific components of an experiment. However, visually analyzing the difference between an SCR and other factors like drift (artifact) can be difficult. Previous research has reported high inter-subject variability in time-domain measures of EDA, particularly in SCL and NSSCRs, which exhibited higher coefficients of variation and lower degrees of consistency between subjects undergoing cognitive, postural, and physical stress, compared to spectral indices. The traditional method of counting SCRs to obtain NSSCRs is challenging when EDA measurements are affected by motion artifacts. These artifacts, such as patient movement, temperature fluctuations, and noise, can make it difficult to distinguish real SCRs from artifacts. This limitation hinders the ability to analyze and interpret ambulatory EDA data. Although manual identification and removal of artifacts from EDA data are possible, it is a time-consuming process. Common approaches to automatically deal with noise and motion artifacts in EDA include low-pass filtering (with a typical cutoff frequency of 1 Hz), exponential smoothing, and removing corrupted signal segments using techniques such as spline-based endpoint connection [79].

There are several other possible major limitations to using EDA and ECG measurements for detecting DN and peripheral arterial disease, some of which were not addressed in previous research. External factors like temperature and humidity can influence EDA measurements, resulting in inconsistent results. Internal factors, such as medication and hydration levels, can also affect EDA measurements and the consistency of results with the same stimulus level. Additionally, while EDA was previously thought to represent a uniform change in arousal across the body, in reality, different locations of measurement can lead to different responses. For example, the EDA measured on the left and right wrist is driven by different regions of the brain, providing multiple sources of arousal, which can lead to varying responses based on sweat gland density and underlying sources of arousal. Furthermore, electrodermal responses are delayed by 1–3 s, adding to the complexity of determining the relationship between EDA and sympathetic activity. Operator skill can also play a significant role in the successful application of EDA measures [79, 80].

Moreover, lower frequency HRV measures that consist of sympathetic and parasympathetic dynamics might not be able to differentiate between patients with DN and those with peripheral artery disease, both of which can lead to decreased blood flow to the feet and increase the risk of ulceration. Additionally, lower frequency HRV measures may be influenced by a range of other factors, such as medication use, comorbidities, and lifestyle factors, making it difficult to isolate the impact of a specific condition [81].

This highlights the need for further research to develop more sophisticated and nuanced measures of HRV that can be used to more accurately assess the risk of FUs in diabetic patients and to monitor the progress of treatment in order to use EDA indices in clinical and everyday settings.

### Recent advances in the use of advanced signal processing for detection and management of diabetic neuropathy and peripheral arterial disease

Posada-Quintero and Chon, in their systematic review [79], also emphasize the progress made in the collection and processing of EDA data. The constant-amplitude AC-voltage exosomatic method has been shown to be the superior approach for collecting reliable EDA data as it avoids complexities and errors introduced by other methods. This method allows for the collection of skin potential, conductance, and susceptance at the same site, making it of interest for those studying the sympathetic nervous system. EDA can be used in conjunction with HRV to develop indices of autonomic function, such as adjusting spectral analysis bands during exercise. In the future, a multi-parameter approach incorporating wearable sensors and artificial intelligence may be used to assess diseases affecting the autonomic control, such as stress and neuropathies. Haque et al. proposed a machine-learning model using electromyography and gait information to detect DN and peripheral artery disease [82].

Artificial intelligence (AI)-based technologies have also been developed to improve remote monitoring of diabetic foot ulcers through mobile apps, which is expected to greatly impact diabetic foot ulcer care. The use of advanced technologies, particularly AI, has been increasing in recent decades to enhance human work output and improve the medical field. AI and its applications are being explored in the context of diabetic foot ulcer care as well, with diabetic foot ulcer datasets being utilized for training and testing AI algorithms. These AI algorithms are developed by using large image datasets from foot clinics, and the process involves several stages, such as pre-processing, feature extraction, detection, classification, and wound segmentation. The main challenge of using AI in real-world settings is overcoming the low-quality images due to various factors, such as inadequate focusing, motion artifacts, and deformities in the foot/toes [83].

Machine learning algorithms have demonstrated promising results in detecting diabetic foot ulcers with high accuracy. The next step in refining the AI algorithms is incorporating classification systems for diabetic foot ulcers to improve AI-based diagnostics and prognosis. Currently, manual diabetic foot ulcer classification systems are being used by foot care professionals and these manual methods may benefit from automation through the use of AI. Overall, the use of advanced signal processing and AI has the potential to greatly improve the detection and monitoring of DN and peripheral arterial disease, as well as other conditions that affect the autonomic control.

## Conclusion

The main purpose of this literature review was to provide an integrated, synthesized overview of the current knowledge of the diabetic foot disease development and the main pathophysiological factors that lead to its onset. EDA and HRV measurements in diabetic patients demonstrate a great potential for early detection of peripheral skin lesions. Moreover, measurements of EDA and HRV could be easily applied in clinical practice. In the future, it would be relevant to conduct larger clinical studies to investigate how EDA and HRV change with the progression of DM and whether there are statistically significant differences in EDA and HRV parameters between healthy subjects and patients recently diagnosed with DN of the feet. It would also be relevant to check whether EDA and HRV values differentiate between different age groups of patients.
